# Case report: Mixed hydrofluoric and nitric acid mist inhalation poisoning

**DOI:** 10.3389/fmed.2024.1342212

**Published:** 2024-10-30

**Authors:** Tongyue Zhang, Xingbing He, Chen Wang, Zhiqiang Zhou, Aerbusili Genjiafu, Zixin Wen, Xiangdong Jian, Qilu Li

**Affiliations:** ^1^Department of Poisoning and Occupational Diseases, Emergency Medicine, Qilu Hospital of Shandong University, Cheeloo College of Medicine, Shandong University, Jinan, China; ^2^Department of Emergency Medicine, Ankang Central Hospital, Ankang, Shanxi, China; ^3^School of Nursing and Rehabilitation, Cheeloo College of Medicine, Shandong University, Jinan, Shandong, China; ^4^Department of Pharmacy, The Hospital of Shandong University, Cheeloo College of Medicine, Shandong University, Jinan, Shandong, China

**Keywords:** mixed acid fog, chemical pneumonia, treatment, poisoning, radiologic changes

## Abstract

**Introduction:**

Hydrofluoric and nitric acids are strongly acidic substances with strong oxidation and are widely used in industrial production. Chemical pneumonia caused by the inhalation of strong acids has occasionally been reported. Severe chemical pneumonia can lead to acute pulmonary edema and life-threatening acute respiratory distress syndrome. The treatment of chemical pneumonia mainly relies on symptomatic and supportive treatment such as anti-inflammatory and anti-infection.

**Case description:**

This study reports three cases of chemical pneumonia caused by the inhalation of a mixed hydrofluoric and nitric acid mist during an occupational exposure accident. After appropriate emergency symptomatic treatment, such as oxygenation and excretion promotion, cough, and difficulty in breathing were alleviated but not completely cleared.

**Discussion:**

Most of the literature reports damage caused by hydrogen fluoride on the skin and mucosal burns, and reports on lung damage caused by the inhalation of large amounts of hydrogen fluoride gas are relatively rare. Usually in the early stage of this kind of patients can have elevated blood picture, hypoxemia, pulmonary CT inflammatory plaque changes, so as to have obvious respiratory irritation symptoms. When inhaling a large amount of hydrofluorine mixed nitric acid mist, in addition to considering the occurrence of severe pneumonia, we should also be alert to the occurrence of other organ damage and electrolyte disorder.

**Conclusion:**

As occupational hydrogen fluoride gas inhalation poisoning often occurs in groups, improving the safety facilities and standard operations of small- and medium-sized enterprises is necessary. For medical workers, it is necessary to be alert to the occurrence of severe toxic pneumonia and severe fluorosis.

## 1 Introduction

Hydrofluoric acid (HF) and nitric acid (HNO_3_) are both highly corrosive chemicals that can destroy human skin and cause strong irritation to the respiratory tract (1–3). They are common sources of chemical damage in industrial production in China, usually caused by skin contact, but also caused by respiratory inhalation (4). In early 2023, a rare poisoning incident of mixed acid mist inhalation of hydrofluoric acid and nitric acid occurred in a factory in Shandong Province. Three patients were diagnosed with poisoning, and after standard treatment in our department, all patients achieved clinical cure. The following is a detailed report.

## 2 Case description

### 2.1 General information

In January 2023, 3 cases of workers in a sealing industrial equipment factory in Shandong Province, China were admitted to our department. The patients were all female and ranged in age from 36 to 50 years with a mean age of 43 years. The average length of service of the three patients was (4 ± 2.27) months. All patients were previously healthy. All patients were married and had a history of childbearing. All three patients had a clear history of mixed acid mist inhalation of hydrofluoric acid and nitric acid in this event. Normally, Three patients worked for 6 h per shift with cumulative exposure to brown-red smoke of 2 h per shift for 20–25 days per month.

### 2.2 Occupational contact history

**Figure 1:**
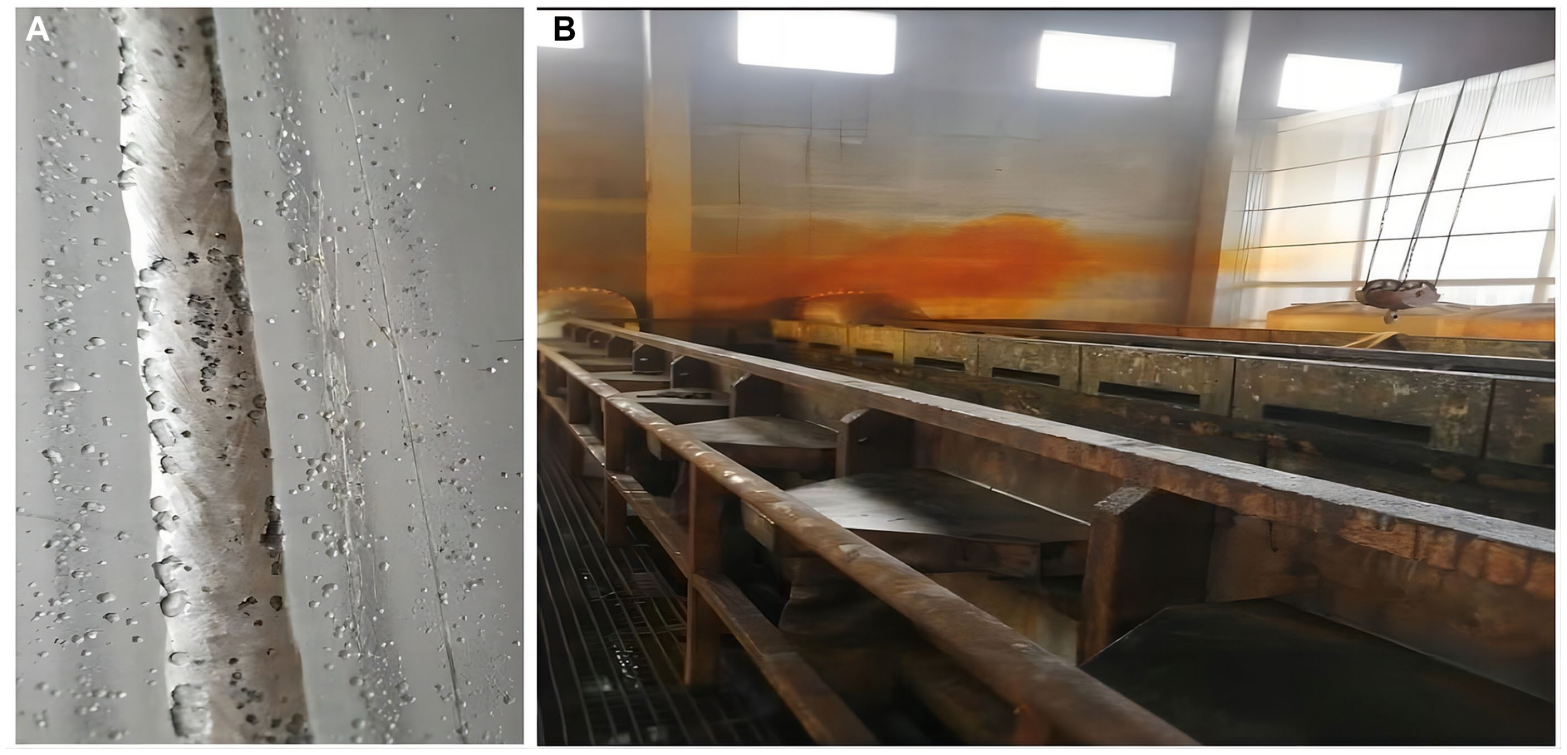
(A) Steel pipe after pickling with hydrofluoric acid and nitric acid mixture. (B) In the production workshop at the time of the accident, brown-red acid fog can be seen.

The sealing industrial equipment factory is located in Shandong Province, China(N36°48′, E118°02′), and mainly produces stainless steel pipes. Stainless steel pipes are widely used in hardware kitchenware, shipbuilding, petrochemical, machinery, medicine, food, power, energy, aerospace, building decoration, and other industries. All patients were operators in the same workshop and shift. The workshop of the three patients was 25 m × 15 m, the pickling pool was 13 m × 1.8 m × 2 m, and there were two exhaust ports, each 0.4 m × 0.4 m. Before pickling, workers open the valves of hydrofluoric and nitric acid containers, put the pickling liquid into the pickling pool, and then use a crane to put 15 m × 1 m stainless steel pipe into the pool. The reaction time varies according to different stainless-steel pipe materials. When the surface of the steel pipe is smooth, the steel pipe is washed with clean water. A small amount of brown-red smoke generated during the pickling process is discharged through the exhaust pipe device. The soaking material used in this accident was a 304 stainless steel pipe, and The patients in this shift entered the workshop 15 h after the material had reacted, and when they were ready for the next operation, a large amount of reddish-brown gas began to appear in the reaction pool. The three patients showed symptoms of discomfort in about 10–20 min, and then left the workshop. It is worth noting that during this accident, red smoke permeated the entire pickling workshop, whereas, in the past, it was mainly concentrated in the exhaust port area. As shown in Figure 1, workers in the production process wear only ordinary masks and do not wear any special personal protective equipment.

## 3 Clinical data

In this incident, three patients showed different degrees of respiratory symptoms, including suffocation, cough, and respiratory burning sensation. Two patients complained of nausea, vomiting, and dizziness. One patient presented with generalized weakness. After a short rest, the above symptoms did not significantly improve, so he went to the emergency department of a third class hospital in Shandong Province and was admitted to our department for further treatment within 24 h of the patient’s exposure time.

**Figure 2:**
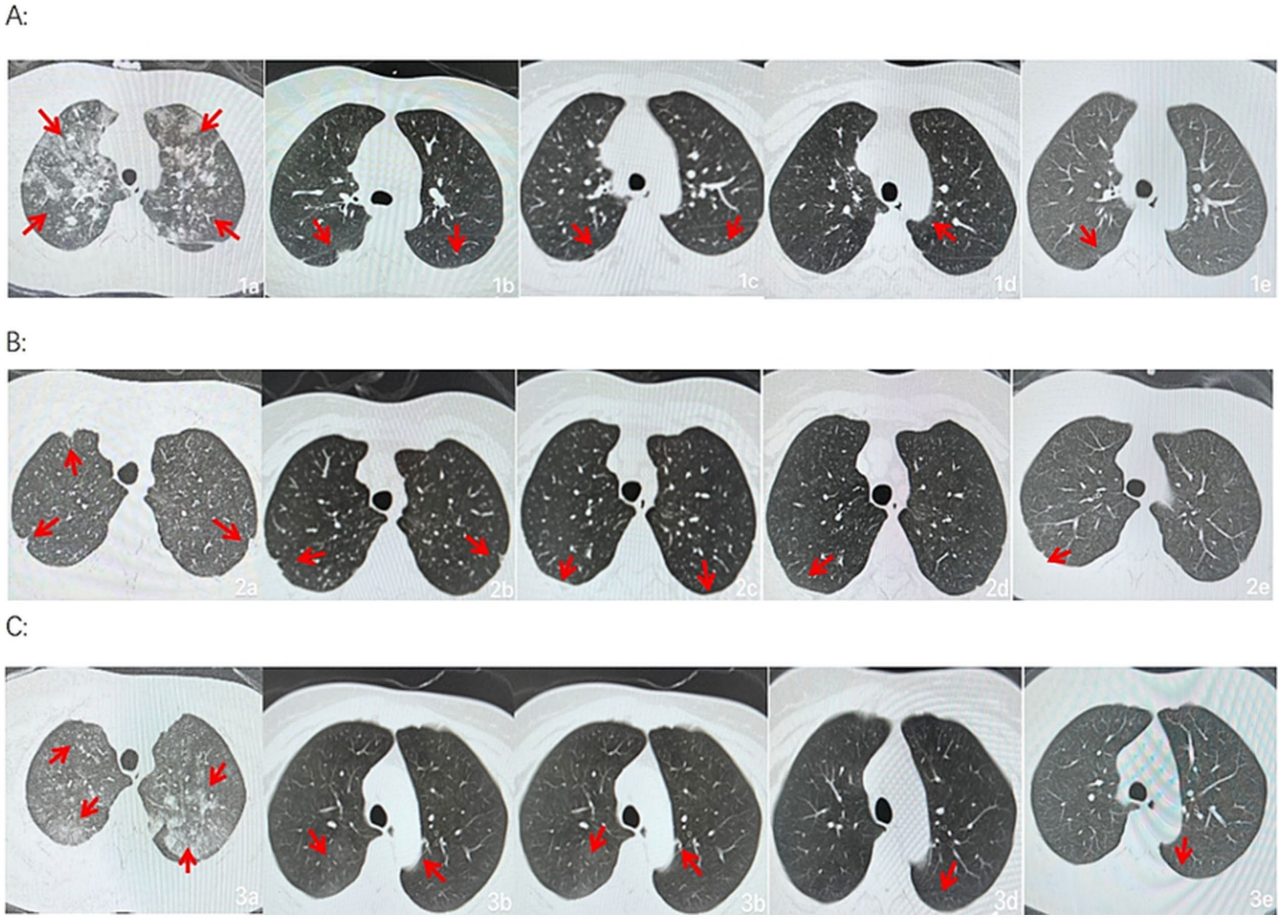
Group A, B and C were from three patients, respectively. a, b, c, d, and e show the changes in bilateral chest CT of Patient at 1, 7, 14, and 21 days after admission and 30 days after discharge, respectively (a, b, c, d, and e are lung windows).

After admission, the three patients underwent laboratory examination (Ausendo 5,600, USA), electrocardiogram (Mindray R12, China), blood gas analysis (cobas 123, China), electromyography (KEYPOINT4, Denmark) and electroencephalogram (EEG-1200c, Japan). Laboratory tests were performed at 3, 7, 14, 21 days after admission and 30 days after discharge, and the changes of white blood cells, neutrophil and other blood counts were closely observed. Chest computed tomography (CT, Definition, Germany) was performed at 1, 7, 14, 21 days after admission and 30 days after discharge to accurately evaluate the imaging efficacy. The laboratory test results of the patients are shown in Tables 1–3. Chest CT is shown in Figure 2. On the first day of admission, Three patients had obvious symptoms of respiratory tract, digestive tract, and eye irritation. Their laboratory test results showed hypoxemia of arterial blood gas, increase of white blood cells and neutrophils, and increased D_2_-polymer in coagulation function. Patients 1 and 3 presented with extensive inflammatory lesions in both lungs, and patient 2 had little inflammation in both lungs. According to the occupational exposure history, clinical manifestations, imaging manifestations and related auxiliary examinations, the three patients were diagnosed as mixed acid fog inhalation poisoning caused by hydrofluoric acid and nitric acid. Because there was no specific antidotic for nitric acid and hydrofluoric acid, the three patients were given symptomatic and supportive treatment during hospitalization, including anti-inflammation, anti-infection, organ protection, prevention of fluorosis, cough relief, reasonable oxygen therapy, etc. The corresponding drugs are methylprednisolone sodium succinate for injection (40 mg/ day, intravenous drip, Huabang Pharmaceutical), flucloxacillin sodium for injection (1 g, 4 times a day, intravenous drip, Taisheng Pharmaceutical), polyene phosphatidylcholine (20 mL/ day, intravenous drip, Tiantaishan Pharmaceutical), calcium gluconate (1 g (10%)/day, intravenous drip, Tiancheng Pharmaceutical), compound Licorice mixture (10 mL, 3 times a day, oral administration, Qilu Pharmaceutical). After 7 days of treatment, the inflammatory response indexes(white blood cells, neutrophils) of the 3 patients were significantly lower than those on admission, the chest CT inflammation was significantly absorbed and improved, the symptoms of cough and suffocation were significantly relieved, and the respiratory burning sensation, tears, dizziness, headache, nausea and vomiting disappeared. Three patients had different treatment duration according to the severity of the disease. The peripheral white blood cells of patients 1 and 3 were slightly increased before discharge, and the rest were within the normal reference range of laboratory tests, reaching the recovery standard: clinical symptoms and signs disappeared. Three patients were reexamined 30 days after discharge, and their respiratory symptoms disappeared, peripheral blood counts were within the normal range, and chest CTs did not indicate pulmonary inflammation. In the whole course of treatment, the liver, kidney and other functional indicators of the 3 patients were within the normal range.

**Table 1:** Main clinical manifestations and examination results of the patients.

Case	Sex	Age	Main clinical manifestation	Radiologic changes
1	Female	43	Cough, dizziness, tearing, limb weakness, suffocation, respiratory burning, nausea and vomiting.	A: double pulmonary plaques and nodular shadows. Combined with medical history, considering toxic changes, bilateral pleural thickening or a small amount of pleural effusion, mediastinal lymph node enlargement; B: The lower lobes of both lungs were spot-like, with bilateral pleural thickening or a small amount of pleural effusion; C: Little inflammation in lower lobe of both lungs; D: There was a little inflammation in the left lung tongue segment. E: double pneumonia was significantly improved compared with before.
2	Female	50	Cough, tearing,chest distress, respiratory burning.	F: bronchiolitis in both lungs, mild chronic inflammation in both lungs, and bilateral pleural thickening; G: Bronchiolitis and a little chronic inflammation in both lungs were absorbed and improved, and local pleural thickening was seen. H: bilateral pulmonary fibrosis, local pleural mild thickening; I: a few fibrous foci in bilateral lungs and mild local thickening of bilateral pleura; J: relief of double pneumonia.
3	Female	36	Cough, phlegm, tears, suffocation, respiratory burning, dizziness, headache, nausea and vomiting.	K: extensive inflammatory lesions in both lungs with a small amount of pleural and pericardial effusion;L: bilateral lung lesions, significantly improved absorption, a small amount of pericardial effusion; M: mild fibrosis and small nodules in both lungs; N: mild fibrosis and small nodules in both lungs, similar to before; O: significant relief of pneumonia in both lungs.

(A, F, K) are CT results of patients on the first day of admission; (B, G, L) are the CT results of patients on the 7th day of admission; (C, H, M) are the CT results of patients on the 14th day of admission; (D, I, N) are the CT results of patients on the 21st day of admission; (E, J, O) are CT results of patients on the 30th day of discharge.

**Table 2:** Laboratory checks for dynamic changes.

Case	Time	WBC (x10^9^/) 3.5–9.5	NEU (%) 31–70	ALT (IU/L) 7–40	AST (IU/L) 13–45	TBIL (μmol/L) 5–21	Cr (μmol/L) 46–106	CK (U/L) 38–174	LDH (U/L) 120–230	DD-i (μg/mL) <0.5	Ca (mmol/L) 2.11–7.52	Mg (mmol/L) 0.65–1.10
1	Day1	**15.39**	**94.5**	14	29	9	55	68	**282**	**0.7**	2.02	0.81
	Day3	**13.61**	**82.8**	11	19	12.9	74	35	**291**	**1.18**	2.16	0.97
	Day7	**11.62**	66	11	15	7.1	67	30	222	**1.11**	2.15	0.92
	Day14	**13.09**	56.3	16	17	9.2	67	26	**231**	**0.56**	2.19	0.94
	Day21	**9.82**	61.7	15	13	9.5	66	36	226	0.39	2.29	0.94
	Day 30 of discharge	4.1	39.5	18	12	8.2	62	84	**238**	0.31	2.31	0.90
2	Day1	7.6	**86.4**	13	12	13	38	29	180	0.17	2.32	0.83
	Day3	9.11	**80.5**	7	11	10.8	51	16	130	0.28	2.28	0.93
	Day7	**10.79**	**74.2**	25	17	11.1	53	18	150	0.3	2.43	1.05
	Day14	**14.9**	68.4	18	14	8.9	45	18	127	0.34	2.23	0.86
	Day21	7.17	56.4	11	10	20.1	53	19	155	0.12	2.45	0.93
	Day 30 of discharge	5.64	52.6	12	21	12.1	45	32	171	0.17	2.40	0.84
3	Day1	**24.95**	**93.2**	15	23	10	53	51	224	**1.46**	2.15	0.97
	Day3	**18.49**	**87.2**	8	10	7.8	59	31	208	**1.8**	2.15	1.07
	Day7	**19.41**	**75.7**	70	22	5.8	65	23	175	**1.01**	2.35	1.09
	Day14	**13.77**	57.5	26	13	5	55	25	131	**0.77**	2.17	0.93
	Day21	**15.17**	62.3	21	10	7.6	59	25	177	**0.69**	2.36	0.93
	Day 30 of discharge	8.3	57.1	8	15	4.3	50	63	173	**0.71**	2.28	0.84

White blood cells, WBC; Neutrophil ratio, NEU%; Glutamic-pyruvic transaminase-ALT; Glutamic oxaloacetic transaminase, AST; Total bilirubine, TBIL; Creatinine, Cr; Creatine kinase, CK; Lactate dehydrogenase, LDH; D-dimer -DD-i; Calcium, Ca; Magnesium, Mg. Outliers in bold.

**Table 3:** Results of arterial blood gas analysis of the patients on admission.

Case	pH (7.35–7.45)	PaO2 (83–108 mmHg)	PCO2 (32–48)	BE (−3–3)	Lac (1.0–1.8 mmol/L)
1	**7.33**	**58.9**	55.8	2.09	1.4
2	7.38	**81.2**	45.1	1.15	1.3
3	7.42	**79.4**	40.2	1.07	1.3

PaO2, Partial pressure of oxygen; PCO2, Partial pressure of carbon dioxide; BE, Base excess; Lac, Lactate acid. Outliers in bold.

## 4 Discussion

Hydrofluoric acid, an aqueous solution of hydrogen fluoride gas, is a colorless, smoky, and corrosive liquid with a sharp, pungent odor. It is an extremely corrosive weak acid and strongly corrodes metals, glass, and silicon-containing materials. Hydrogen fluoride stimulates and corrodes the skin, eyes, respiratory tract, and gastrointestinal mucosa. Fluoric acid can cause obvious burns at the contact site and dehydrate and dissolve histamine. It can quickly penetrate the stratum corneum and deep tissues, dissolve cell membranes, and cause tissue liquefaction. The inhalation of high-concentration steam or percutaneous absorption can cause chemical pneumonia and pulmonary edema (5, 6). Nitric acid is soluble in water; a pure nitric acid solution at room temperature is colorless and transparent, and its physical properties are unstable and easily decompose with light or heat, producing nitrogen dioxide, which when dissolved in nitric acid, produces a light yellow appearance. The mechanism of acute lung injury caused by nitric acid gas is believed that first, nitric acid gas directly damages the respiratory mucosa, and the other is the interaction of various complex mechanisms such as inflammatory response, respiratory burn-oxidative stress, neuroregulation, and chronic injury (7). Although the former is a weak acid and the latter is a strong acid in physical properties, when a large amount of hydrofluoric acid and nitric acid are inhaled, they will directly damage the respiratory mucosa tissue and alveolar surfactant, cause inflammatory response of the body, and cause eye, respiratory tract, digestive tract irritation response and lung inflammation. There is no significant difference in clinical manifestations and imaging changes. In particular, hydrofluoric acid is more permeable to tissue damage. In addition, fluoride ions in the blood or tissue can combine with calcium and magnesium ions and become insoluble or slightly soluble calcium fluoride and magnesium fluoride; in severe cases, it directly blocks blood vessels and even affects the function of the central nervous and cardiovascular systems, resulting in hypocalcemia and hypomagnesemia syndrome, fluoride ions can also combine with hemoglobin to form fluoroheme, inhibit succinate dehydrogenase, and prevent fluoride ions development (8). Decreased oxygenation affects cellular respiratory function. Hydrofluoric acid burns combined with fluorosis should be paid attention to. Patients may have convulsions due to hypocalcemia, prolonged Q-T interval of electrocardiogram, and ventricular fibrillation attack (9).

When nitric and hydrofluoric acids are mixed, nitric acid acts as a strong oxidizing agent and hydrofluoric acid as a complexing agent; the two have a strong oxidation–reduction reaction to produce hydrogen fluoride and nitrogen dioxide and generate heat simultaneously. Moreover, nitric acid itself can also produce nitrogen oxides under heat and light. Nitrogen oxide is insoluble in water and can penetrate the distal lungs bronchioles and the adjacent alveolar tissues, causing lung damage. The chemical damage caused by nitrogen oxides has a long incubation period, and the latency time varies from a few hours to several days. Initially, it can manifest as cough, sputum, mild dyspnea, and other mild symptoms; however, the condition may suddenly deteriorate, manifesting as severe dyspnea, tracheal spasms, pulmonary edema, and respiratory distress syndrome. Therefore, patients exposed to such irritating gases require early treatment, attention to disease changes, and a reduction in serious complications (10).

In this case, the patients had respiratory discomfort such as difficulty in breathing, cough, and burning sensation of the respiratory tract from initial contact, and no redness, swelling, or chemical burns were observed in the skin mucosa of the oropharynx, nose, eyes, or limbs, consistent with respiratory irritation symptoms caused by the inhalation of hydrofluoric and nitric acids. After appropriate emergency symptomatic treatment, such as oxygenation and excretion promotion, cough, and difficulty in breathing were alleviated but not completely cleared. Considering the literature reports on large-scale lung inflammation and even acute respiratory distress syndrome after inhaling a considerable number of irritating gases, chest CT examination was completed upon admission of the patients, which showed that one patient had extensive inflammatory lesions in both lungs, one had bronchitis changes in both lungs, and one had plaques and nodules in both lungs. All three were normal after pre-service physical examination. The patients worked in the same environment, and varying degrees of lung disease severity are considered to be related to individual sensitivity and protection. After the patients were treated with anti-infection and anti-inflammatory treatment for 1 week, chest CT showed that their lung lesions significantly improved. During hospitalization, the patients’ symptoms gradually stabilized under anti-inflammatory, anti-infection, metabolic promotion, nutritional support, and other treatments, and inflammation gradually decreased without pulmonary edema, tracheal spasms, or other disease progression. The patients were re-examined 30 days later and were in good physical condition to achieve a clinical cure. It follows that although there is no specific cure for nitric acid and hydrofluoric acid poisoning, the general principles of acute poisoning apply to the treatment of pneumonia caused by inhalation of chemical gases, and are remarkably effective in preventing complications and improving prognosis.

In addition to that, most of the literature reports damage caused by hydrogen fluoride on the skin and mucosal burns, and reports on lung damage caused by the inhalation of large amounts of hydrogen fluoride gas are relatively rare. Therefore, severe pneumonia should be considered in cases of inhalation of large amounts of mixed hydrofluoric with nitric acid mist (11, 12). Notably, intravenous calcium gluconate supplementation is emphasized in the treatment of patients with hydrogen fluoride burns to correct hypocalcemia and hypomagnesemia and prevent serious cardiovascular damage (13). Blood gas analysis and blood biochemical electrolyte examination were performed upon admission on the three patients in this case. Although only one patient’s calcium ion was slightly lower than the normal reference value on the first day of admission, intravenous calcium gluconate injection was routinely administered to the patients to prevent delayed electrolyte disturbances caused by invisible, hidden burns and possible malignant events such as psychological disorders and cardiac arrest. Blood gas and electrolyte levels were monitored later, and the patient’s test results were normal (14).

As occupational hydrogen fluoride gas inhalation poisoning often occurs in groups, workers in these enterprises usually lack knowledge and experience in occupational disease prevention and control, and their awareness of protection is weak. Most of the workers did not wear personal protective equipment in accordance with the regulations, and this incident was caused by the patients not being strictly equipped with protective equipment. The production process is relatively primitive, resulting in the inhalation of a large amount of acid mist. Therefore, improving the safety facilities and standard operations of small- and medium-sized enterprises is necessary (15, 16). In industrial production, it is still necessary to strengthen the training of pre-job personal protective measures for workers, clarify the risk of hydrofluoric acid, nitric acid and other acidosis in production, increase personal safety awareness, improve the corresponding protective facilities, identify dangerous chemicals, strengthen the supervision of hydrofluoric acid and nitric acid standard use, and improve the emergency plan for acute poisoning (17–20). The deficiencies of this event include the absence of on-site air measurement data owing to the urgency of the event and the lack of specific concentrations of nitric and hydrofluoric acids involved in the pickling process.

## 5 Conclusion

Occupational gas inhalation injury is not rare in production work, but the cases of pneumonia caused by gas inhalation injury are rarely reported. Therefore, it is found that early prevention and treatment through anti-inflammation, anti-infection, protection of organs, and maintenance of internal environment stability can reduce the serious complications and death of such patients.

## Data Availability

The original contributions presented in the study are included in the article/supplementary material, further inquiries can be directed to the corresponding authors.
